# The joint effects of local, climatic, and spatial variables determine soil oribatid mite community assembly along a temperate forest elevational gradient

**DOI:** 10.1002/ece3.11590

**Published:** 2024-07-03

**Authors:** Dandan Liu, Haitao Wu

**Affiliations:** ^1^ Key Laboratory of Wetland Ecology and Environment, Northeast Institute of Geography and Agroecology Chinese Academy of Sciences Changchun Jilin China

**Keywords:** community assembly, ecological processes, mountains, oribatid mites, spatial and environmental filters

## Abstract

Numerous factors influence mountain biodiversity variation across elevational gradients and recognizing the relative importance is vital for understanding species distribution mechanisms. We examined oribatid mites at nine elevations (from 600 to 2200 m a.s.l) and four vegetation types from mixed coniferous and broad‐leaved forests to alpine tundra on Changbai Mountain. We assessed the contribution of environmental factors (climatic and local factors) and spatial processes (geographic or elevation distances) to oribatid mite community assembly and identified 59 oribatid mite species from 38 families and 51 genera. With increasing elevation, species richness and the Shannon index declined significantly, whereas abundance followed a hump‐shaped trend. Soil TP, NH_4_^+^‐N, MAT, MAP, and elevation were the critical variables shaping oribatid mite communities based on random forest analysis. Moreover, environmental and spatial factors, and oribatid mite communities were significantly correlated based on Mantel and partial Mantel tests. Local characteristics (3.9%), climatic factors (1.9%), and spatial filtering (8.8%) played crucial roles in determining oribatid mite communities across nine elevational bands (based on variation partitioning analyses of abundance data). Within the same vegetation types, spatial processes had relatively little effects, with local characteristics the dominant drivers of oribatid mite community variation. Environmental and spatial filters together shape oribatid mite community assembly and their relative roles varied with elevation and vegetation type. These findings are crucial for the conservation, restoration, and management of Changbai mountain ecosystems in the context of climate change, along with the prediction of future vertical biotic gradient pattern evolution.

## Graphical abstract

Soil oribatid mites exhibited significant variation along an elevational gradient and within different vegetation zones. The assembly of oribatid mite communities along elevational gradients is dominated by dispersal limitation. Elevation changes significantly influence the assembly of oribatid mite communities through environmental filters.

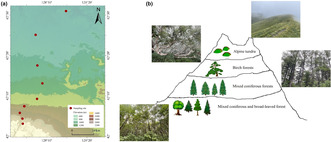

## 1 INTRODUCTION

Mountain systems harbor an evolutionarily unique and exceptionally large portion of global biodiversity (Antonelli et al., 2018; Rahbek, Borregaard, Antonelli, et al., 2019; Rahbek, Borregaard, Colwell, et al., 2019). Along elevational gradients, environmental conditions vary immensely over small spatial scales, creating starkly different habitats and climatic zones (Montaño‐Centellas et al., 2021; Perrigo et al., 2020; Wang, Hu, et al., 2022; Wang, Zhong, et al., 2022). Such variations lead to one of the most frequently documented relationships: the elevation‐diversity connection (Graham et al., 2014; Wang, Hu, et al., 2022; Wang, Zhong, et al., 2022). Historically, the belief was that diversity either declines linearly with increasing elevation or peaks at mid‐elevations, mirroring latitudinal shifts (Rahbek, Borregaard, Antonelli, et al., 2019). Given these intricate dynamics, disentangling the mechanisms underlying biodiversity generation and maintenance is the key to determining why these differences exist (Zhang et al., 2020; Zhou & Ning, 2017). One approach lies in understanding community structures and ecosystem processes that shift along elevational gradients (Körner, 2000). Indeed, mountain biodiversity patterns are driven by many factors, including climate, geography, and elevation, among others, but there is no current consensus on their interplay (Graham et al., 2014).

Delving deeper into mountain systems, below‐ground biodiversity emerges as a critical, yet often overlooked component (Korboulewsky et al., 2016), as diverse soil biota, arguably have more profound implications for ecosystem function than their above‐ground counterparts (Gongalsky, 2021; Liu et al., 2019). Given their diminutive organism size and vast diversity, mechanisms underlying their community structure and biogeography are generally less understood. A notable member of this underground community is the soil oribatid mite, and in mountain ecosystems, their communities frequently shift with elevation (Hasegawa et al., 2006; Illig et al., 2010; Marian et al., 2019; Mumladze et al., 2015). Previously, we investigated oribatid mites at nine elevations (from 600 to 2200 m a.s.l) on Changbai Mountain (Liu, Liu, et al., 2023). The average oribatid mite density was 77,000 individuals per m^2^, and species abundance showed a hump‐shaped distribution along the elevational gradient. While previous studies have shed light on some patterns and influential factors, many questions still linger. For instance, we have discerned that local habitat complexity and climatic shifts play significant roles (Liu, Liu, et al., 2023; Liu, Wu, et al., 2023), but a comprehensive understanding remains elusive.

Understanding spatial biodiversity patterns along environmental gradients is a pivotal research area in ecology (Montaño‐Centellas et al., 2021; Ning et al., 2019). The traditional niche‐based theory (deterministic processes), hypothesizes that deterministic factors, like species traits and interspecies interactions, govern community structure (Fargione et al., 2003). Conversely, the neutral theory assumes that community structures result from more random processes rather than species‐specific attributes (Zhou & Ning, 2017). Empirical research has further underscored the role of abiotic factors as primary determinants in shaping species richness and abundance in mountain ecosystems (Boucher‐Lalonde et al., 2014). At high elevations, inhospitable conditions may lead to depauperate assemblages due to intense environmental filtering, which restricts both species persistence and colonization (Graham et al., 2014). Conversely, mid‐elevation zones may have increased biodiversity, attributable to the presence of wide‐ranging species and elevated productivity (McCain, 2009; Quintero & Jetz, 2018). Yet, despite these insights, there is still much to uncover about the specific processes at play in mountain systems.

The role of spatial processes in influencing soil faunal biodiversity has gained recent attention (Dirilgen et al., 2018; Liu et al., 2019). Dispersal is a pivotal factor in an organism's life history, influencing soil fauna distribution at various scales (Bonte & Dahirel, 2017; Liu et al., 2019). Nevertheless, soil fauna typically disperses only a few centimeters through the soil matrix, indicating that broader geographic and elevational dispersal significantly affects soil fauna distribution. Theory predicts that environmental filtering becomes a dominant force at higher elevations, imposing constraints that hinder species survival (Graham et al., 2014; Montaño‐Centellas et al., 2021). Such filters can lead to species reductions at high elevations due to harsh environmental conditions, like lower temperature and productivity (Montaño‐Centellas et al., 2021). Factors influencing soil fauna distributions may vary spatially, with soil properties playing a significant role across all scales (Devetter et al., 2017; Liu et al., 2019; Xie, Sun, et al., 2022). Moreover, both geographic and environmental filtering should be considered integrative factors, initially impacting vegetation and soil properties, which subsequently influence soil invertebrate inhabitants.

Based on previous research on soil mesofauna communities along elevation gradients in the Changbai Mountain, we observed a decline in species diversity at higher elevations due to harsh environmental conditions, including low temperatures, intense ultraviolet radiation, and sparse vegetation (Liu, Liu, et al., 2023; Liu, Wu, et al., 2023; Xie, Sun, et al., 2022). Consequently, we infer that the oribatid mite community in the alpine tundra above 2000 m has a simplified composition and low density due to the harsh environmental conditions. Given these insights, we address two main questions: (1) What are the crucial environmental factors shaping oribatid mite communities? and (2) How significant are environmental (local and climatic) and spatial (geographic or elevation distances) factors in structuring oribatid mite communities across nine elevations and four distinct vegetation types on Changbai Mountain? Our hypotheses are as follows: 1. Oribatid mite assemblages are jointly affected by climatic conditions, local habitat features, and spatial filtering; 2. At high elevations, environmental filtering has a greater impact on oribatid mite communities than spatial filtering; and 3. Significant geographic and elevation differences can limit oribatid mite dispersion at larger scales, with little dispersal limitation within the same vegetation type at different elevations.

## 2 MATERIALS AND METHODS

### 2.1 Site description and sampling design

**FIGURE 1:**
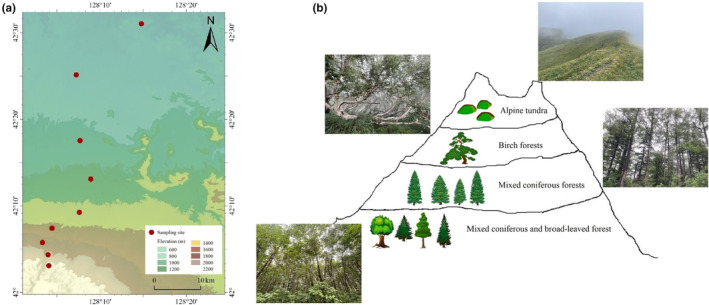
(a) Map of the study area across nine elevations (from 600 to 2200 m) and (b) four vegetation types on Changbai Mountain.

The research site is situated within Changbai Mountain National Nature Reserve (41°41′–42°51′ N, 127°43′–128°16′ E). Changbai Mountain, spanning an elevation from 648 to 2728 m asl, harbors a diverse range of vegetation types reflective of temperate to cold zone transitions in Eurasia. Mixed coniferous and broad‐leaved forests occur up to 1100 m, mixed coniferous forests between 1100 and 1700 m, birch forests between 1700 and 2000 m, and alpine tundra above 2000 m (Figure 1; Table S1).

Soil samples were collected in July 2021 at 200 m intervals from 200 to 2200 m on Changbai Mountain (Figure 1). Additional study area information can be found in Liu, Liu, et al. (2023); Liu, Wu, et al. (2023). At each sampling location, eight independent replicate plots, each 20 × 20 m and spaced 50–100 m apart, were established at random, and a composite soil sample was collected from each. Each composite sample comprised three discrete soil cores, each with a diameter of 5.6 cm and a depth of 10 cm. In total, 72 soil samples were collected across the nine elevations. All plots were established on north‐facing, similar slopes.

### 2.2 Soil fauna identification and laboratory procedures

Soil fauna was separated using a modified Tullgren funnel method (without heating and extending the separation duration to over 10 days), then preserved in 95% ethanol for subsequent identification. Soil oribatid mites were separated based on morphological characteristics under a stereo‐microscope. Adults were counted and identified to species level using a fluorescence microscope (Nikon 80i) for soil oribatid mites (Balogh & Balogh, 1992; Krantz & Walter, 2009; Ryabinin et al., 2018; Yin et al., 2000).

**FIGURE 2:**
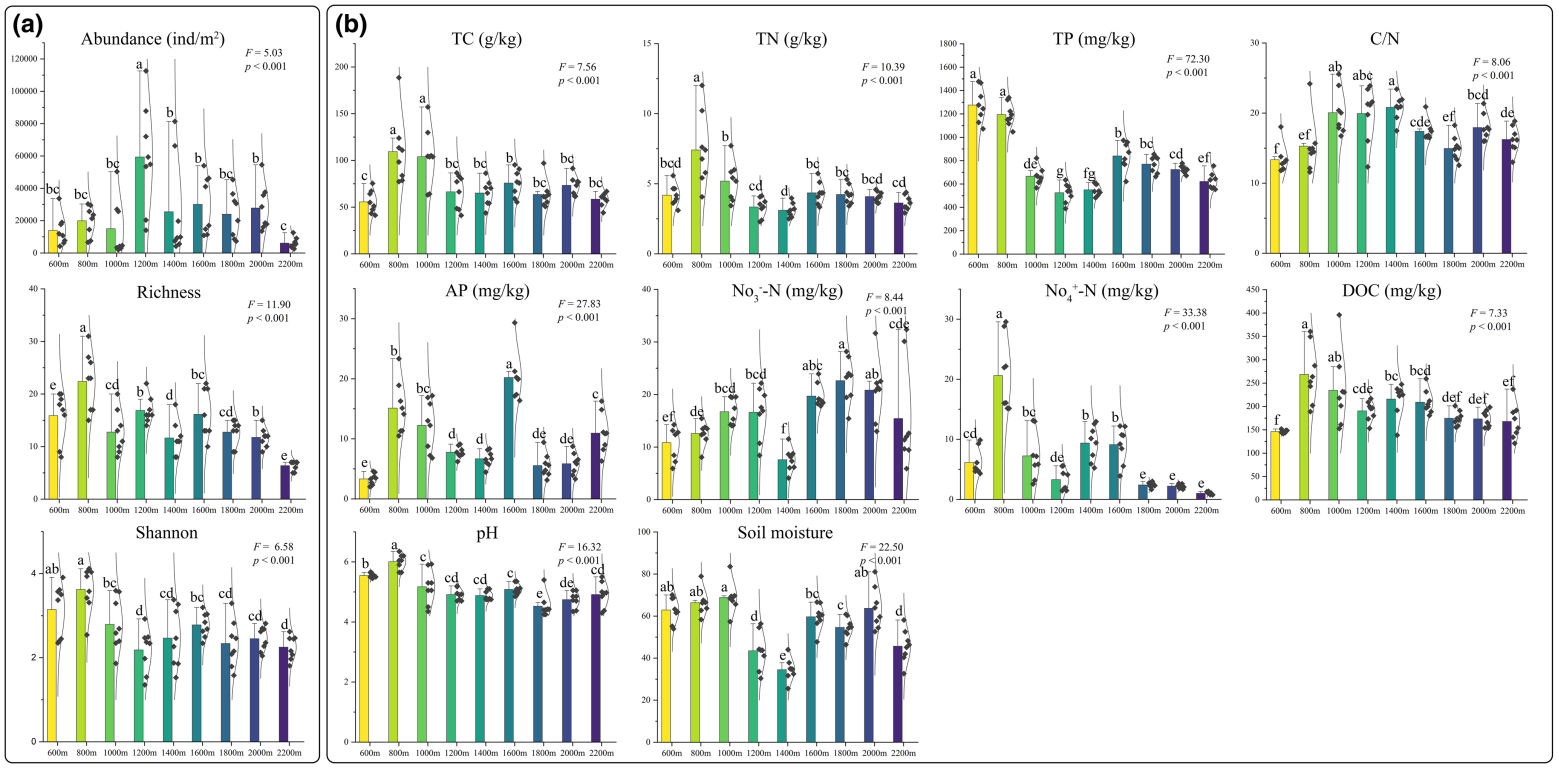
The means of soil oribatid mite diversity (a) and soil physicochemical properties (b) across nine altitudinal gradients. Error bars are standard errors and different letters indicate significant differences (Tukey HSD) between elevations.

The three soil cores from each plot were amalgamated, employing a consistent sampling method. Visible roots and rocks were extracted, followed by indoor air‐drying and pulverization through a 100‐mm mesh sieve. This process was executed to facilitate subsequent analyses for various soil physicochemical parameters, including soil pH, total organic carbon (TC), nitrogen (TN) and phosphorus (TP), soil dissolved organic carbon (DOC), ammonium nitrogen (NH_4_^+^‐N) and nitrate nitrogen (NO_3_^−^‐N), available phosphorus (AP), and soil moisture (SM). Further information on methods employed for deriving physicochemical indices can be found in Liu, Liu, et al. (2023) (Figure 2b).

### 2.3 Geographic information and climatic data collection

Sampling site longitude and latitude were recorded at the time of soil sampling. Mean annual temperature (MAT) and mean annual precipitation (MAP) (2019, 2020, and 2021 average) were obtained from the National Meteorological Information Center (http://data.cma.cn). Geographic and climatic data are shown in Table S1.

### 2.4 Community diversity analyses

Species abundance across varying sampling sites was normalized to 1m^2^ for subsequent analyses. We used richness, abundance, and the Shannon index as oribatid mite community biodiversity indicators. Nonmetric multidimensional scaling (NMDS) ordination and permutational multivariate analysis of variance (PERMANOVA) were used to calculate variance in oribatid mite communities based on relative abundance data (Bray–Curtis distance) and presence/absence data (Sorensen distance). NMDS and PERMANOVA analyses were conducted using the “metaMDS” and “adonis2” functions in the “vegan” R software package (Dixon, 2003). Based on the stress values obtained, we built 3D‐NMDS.

### 2.5 Environmental factors and oribatid mite assemblages

Redundancy analysis (RDA), using Monte Carlo permutation with 9999 repetitions, was employed to discern the primary environmental determinants influencing oribatid mite communities, relying on Hellinger's pretransformed community data (Gao et al., 2014). Environmental variables including soil TC, TN, TP, DOC, NH_4_^+^‐N, NO_3_^−^‐N, AP, and SM were log‐transformed. Our analysis incorporated both presence/absence and abundance data. While abundance data accentuates the relative prominence of specific species (Declerck et al., 2011), presence/absence data offer insights into distribution patterns and buffers predominant species influence. Most community variation explanatory variables were selected from the assessed environmental factors through forward selection, using the “ordiR2step” function in the R package “vegan” (Blanchet et al., 2008). Furthermore, RDA factored in sample elevation values, considering elevation as a surrogate for unmeasured environmental stressors, such as ultraviolet radiation (Quenta Herrera et al., 2018).

Spearman's rank correlations were used to assess associations between oribatid mite diversity (richness) and environmental variables (MAT, MAP, elevation, TP, NH_4_^+^‐N, pH, TC, TN, SM, AP, NO_3_^−^‐N, C/N, and DOC). To discern the most predictive environmental factors for oribatid mite richness, we conducted a random forest analysis using the “randomForest” package in R. Environmental variable importance was gauged by the percentage increase in the mean squared error (MSE); notably, elevated MSE% values denote paramount variables (Breiman, 2001). Model significance and cross‐validated *R* ^2^ values were evaluated through 5000 permutations of the response variable using the “A3” package. The significance of each predictor on the response variables was assessed with the “rfPermute” package (Jiao et al., 2018).

### 2.6 Spatial variables and oribatid mite assemblages

To evaluate the impact of spatial variables on oribatid mite community structure, we conducted a principal coordinate of neighbor matrices (PCNM) analysis across the nine elevations, based on geographic coordinates and elevation for each soil sampling site. Moreover, across various elevations within the same vegetation type, we performed PCNM analyses of geographic coordinates for each soil sample with varying elevation differences to discern the relative contribution of spatial variables to community variation across four vegetation types. PCNM analysis generates a set of orthogonal spatial variables (PCNMs) that encapsulate spatial variability across multiple scales (Declerck et al., 2011; Dray et al., 2006; Guo et al., 2020). We constructed PCNMs for various spatial scales to delineate spatial patterns within communities and as predictive variables for community variation (Liu et al., 2021). For this analysis, spatial components were computed employing the “PCNM” function in the R package “PCNM” (Declerck et al., 2011).

### 2.7 Quantifying environmental and spatial factor effects

Mantel and partial Mantel tests were conducted using the “vegan” package to assess the influence of spatial and environmental distances on oribatid mite community dissimilarities (Guo et al., 2020). Notably, the geographic distances between sample sites do not fully encapsulate the spatial structure related to species dispersal.

To discern the relative contributions of environmental and spatial processes on the oribatid mite community, we performed variance partitioning analysis (VPA) for the nine elevations and four vegetation types. VPA was based on local (soil properties), climate (MAT and MAP), and spatial variables (PCNMs) partition criteria to show independent or joint effects. Local factors were those identified in the RDA and forward selection, excluding elevation (Figures S1 and S2). MAT and MAP selection as primary climatic variables stemmed from the RDA and random forest analysis findings. The PCNMs, derived from geographic and elevational distances, were considered distinct spatial components (Quenta Herrera et al., 2018). Consequently, we established two models: Model 1 related the oribatid mite community to the environment and geographic coordinate‐based distances; Model 2 related the oribatid mite community to the environment and elevation‐based distances. We used adjusted *R* ^2^ values across our analyses, given their unbiased representation of the explained variation (Peres‐Neto et al., 2006). The presence of significant environmental control with nonsignificant spatial control suggests niche processes. Conversely, significant spatial control with nonsignificant environmental control indicates dispersal limitation. Nevertheless, prudence is necessary when analyzing and interpreting the findings, given the methodological limitations (Logue et al., 2011). All analytical processes were executed in the R “vegan” package.

## 3 RESULTS

### 3.1 Oribatid mite assemblages

**FIGURE 3:**
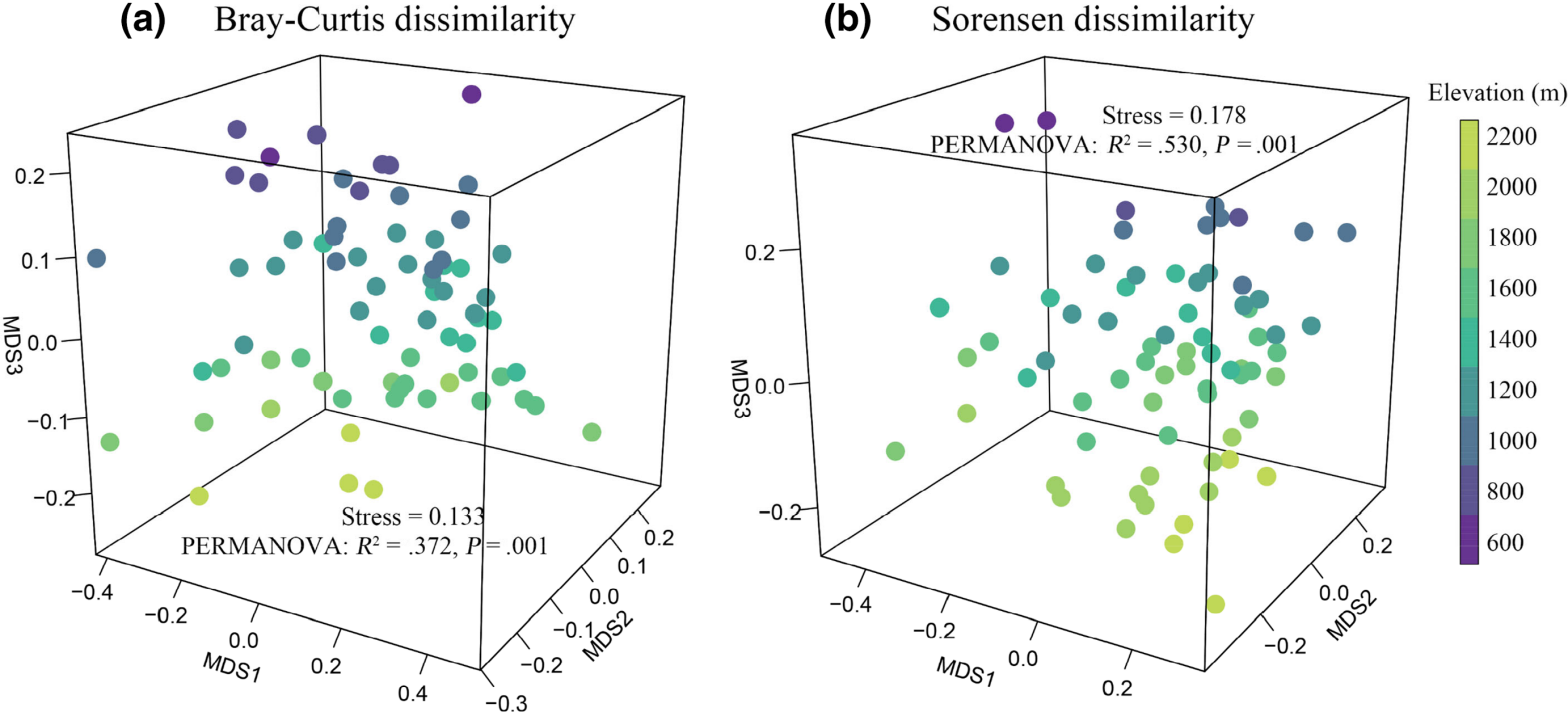
Nonmetric multidimensional scaling (NMDS) ordination plots depicting the distribution of soil oribatid mite communities across nine elevations (*n* = 72). The distance between samples (points) represents distinctness in community composition, calculated as dissimilarity based on relative abundance data (Bray–Curtis, a) or presence/absence data (Sorensen, b).

From a total of 12,948 soil oribatid mites identified, there were 59 species belonging to 38 families and 51 genera (Table S2). Overall, the most abundant species was *Lauroppia neerlandica* (44.78% relative abundance), followed by *Tectocepheus velatus* (14.50%). Common taxa (1%–10% relative abundance) were *Platynothrus peltifer*, *Eniochthonius minutissimus*, and 14 *Trichoribates* sp. (30.29%), whereas 43 species (10.43%) were rare taxa, with a relative abundance of <1%. Oribatid mite abundance showed a hump‐shaped pattern with increasing elevation, reaching a maximum of 1200 m. Species richness and the Shannon index decreased with increasing elevation, with a maximum of 800 m and a minimum of 2200 m (Figure 2a). Oribatid mite community composition significantly differed across different elevations (Bray–Curtis dissimilarity: *R* ^2^ = 0.372, *p* = .001; Sorensen dissimilarity: *R* ^2^ = 0.530, *p* = .001) (Figure 3).

### 3.2 Environmental factors

**FIGURE 4:**
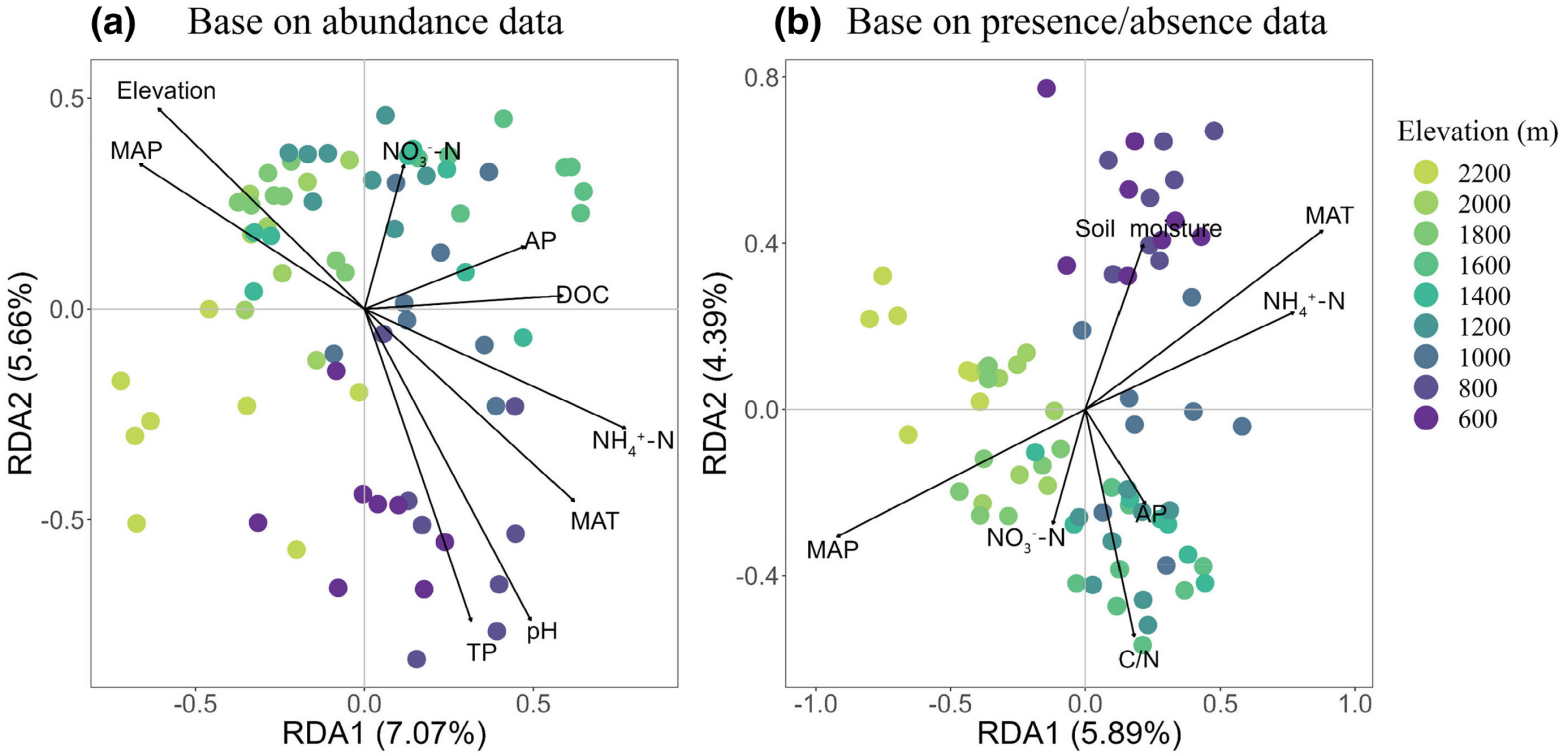
Redundancy analysis (RDA) on Hellinger‐transformed data for oribatid mite communities across nine elevations based on (a) abundance data and (b) presence/absence data.

Nine environmental variables explained community variation, including elevation, TP, AP, NO_3_^−^‐N, NH_4_^+^‐N, DOC, pH, MAP, and MAT (*p* < .05; Figure 4). The RDA using these variables accounted for 12.73% of the total community variance across nine elevations. Specifically, oribatid mite communities at lower elevations (600–1000 m) were exhibited positive correlations with TP, pH, MAT, and NH_4_^+^‐N, while AP, DOC, and NO_3_^−^‐N were positively correlated with oribatid mite communities at mid‐elevations (1200–1600 m) but negatively at 2200 m asl. Both elevation and MAP were positively correlated with oribatid mite communities at 1800 m and 2000 m. Additionally, environmental variables that explained community variation included soil moisture, NH_4_^+^‐N, NO_3_^−^‐N, AP, MAT, and MAP (*p* < .001). RDA with these variables explained 10.28% of the total community variation. We obtained similar results when we omitted elevation as an environmental variable in the RDA (Figure S3). Overall, the RDA explained 13.22% of the community variation using abundance data (*p* < .001) and 10.53% for presence/absence data (*p* < .001; Figure S3).

**FIGURE 5:**
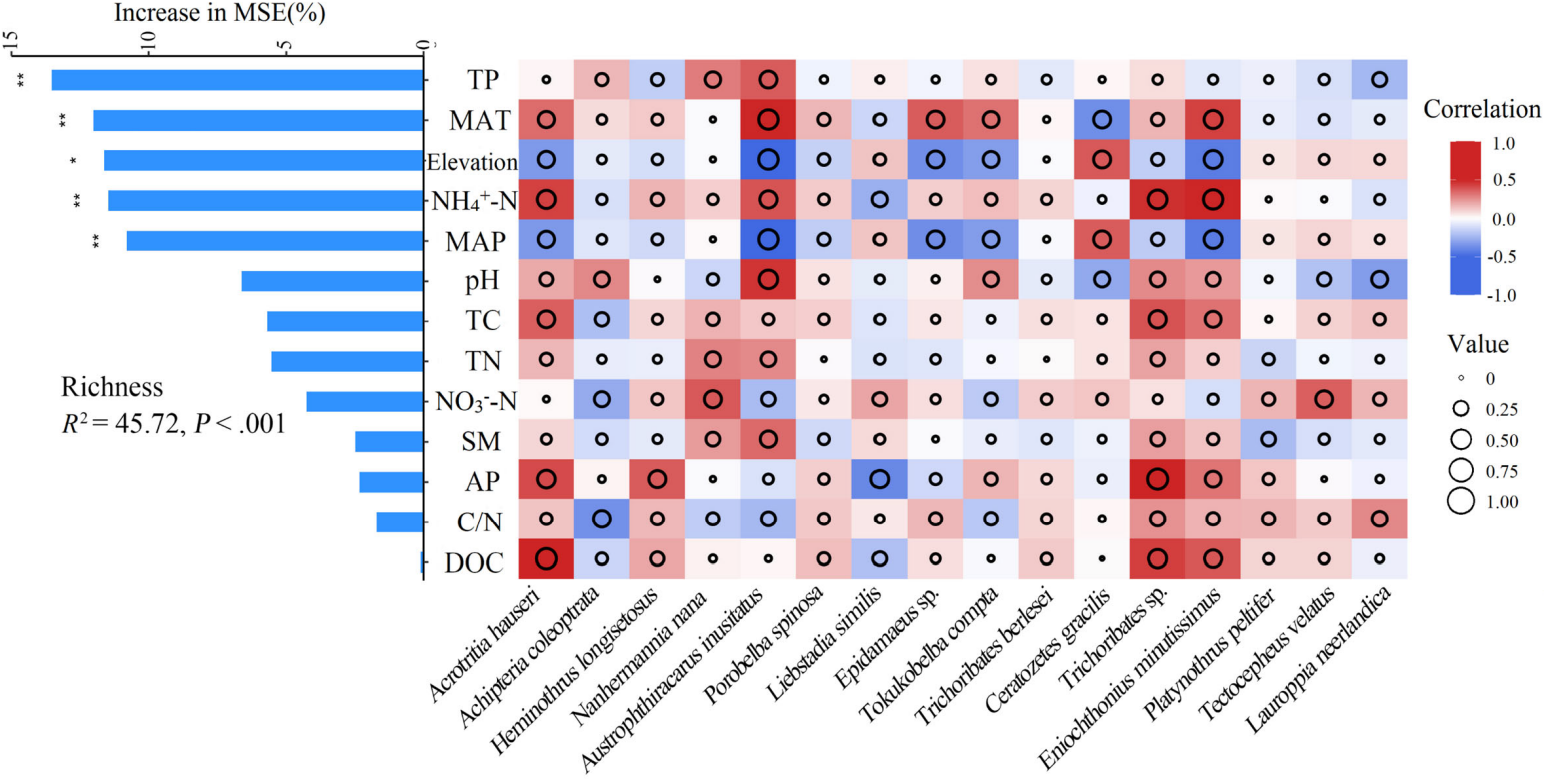
Correlation and random forest model for the relative abundance of dominant oribatid mite species on Changbai Mountain. Random forest (RF) means predictor importance (percentage of increase in mean square error) of environmental factors as drivers for species richness. Circle size represents the absolute correlation coefficient value. Colors represent Spearman correlations.

For the dominant oribatid mite species, the relative abundance of 11 was significantly correlated (*p* < .05) with variables such as pH, elevation, MAT, and MAP (Figure 3; Table S3). Richness, abundance, oribatid mite Shannon index, and environmental variables including soil TP, TC, pH, DOC, elevation, NH_4_^+^‐N, MAP, and MAT were strongly related (Table S4). Moreover, soil TP, NH_4_^+^‐N, MAT, MAP, and elevation were the key drivers of oribatid mite community distribution along the elevational gradient (*R* ^2^ = 45.72, *p* < .001; Figure 5).

### 3.3 Relative importance of environmental and spatial processes

**FIGURE 6:**
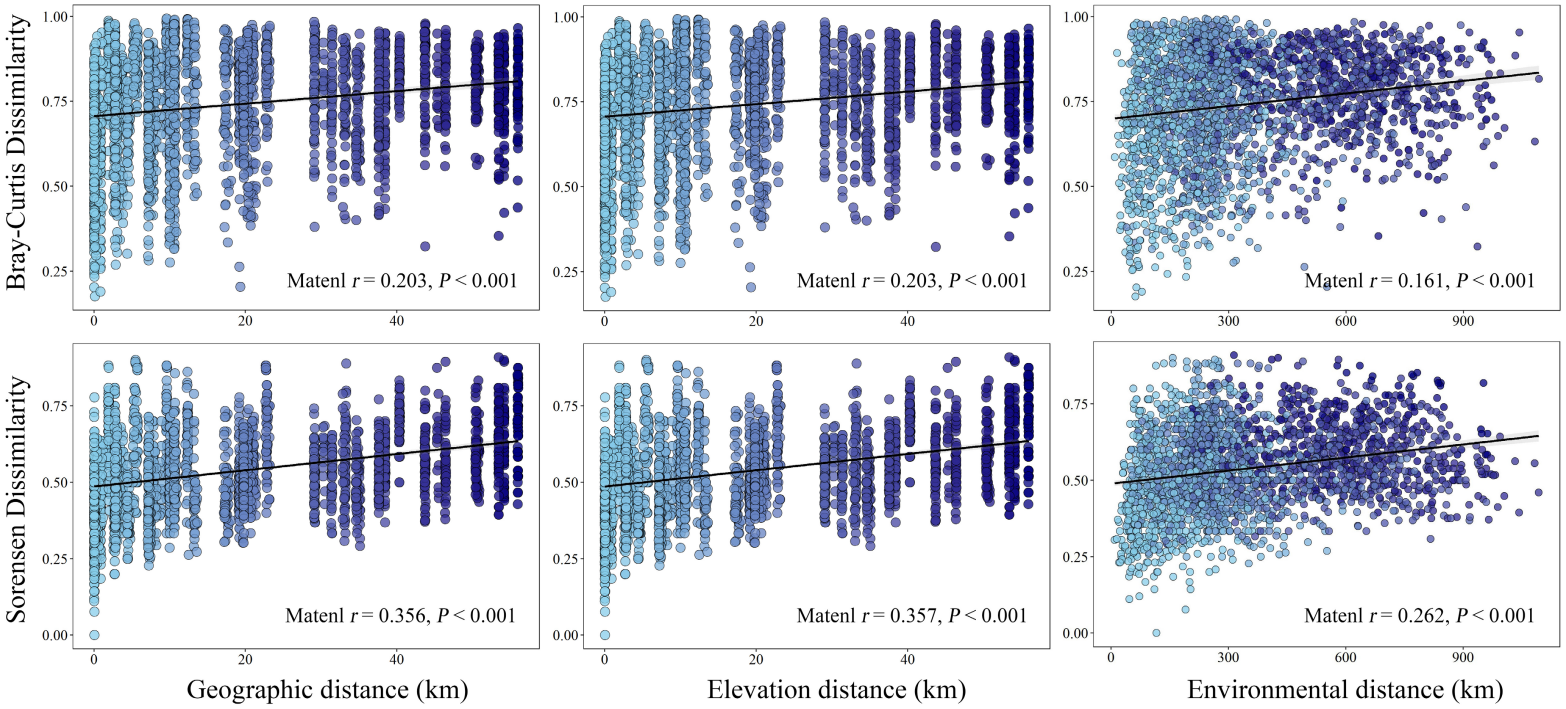
Relationship between geographic distance, elevation distance, and oribatid community dissimilarity (based on Bray–Curtis distances or Sorensen distances) in soil samples. Environmental distance was calculated as scaled Euclidean distance. The solid line represents the fitted linear regression model, assisting in explaining the correlation trends.

Oribatid mite community dissimilarity significantly increased with geographic distance (Bray–Curtis dissimilarity: Mantel *r* = .203, *p* < .001; Sorensen dissimilarity: Mantel *r* = .356, *p* < .001) (Figure 6). Similar results were obtained for elevation distance (Bray–Curtis dissimilarity: Mantel *r* = .203, *p* < .001; Sorensen dissimilarity: Mantel *r* = .357, *p* < .001) and measured environmental distance (Bray–Curtis dissimilarity: Mantel *r* = .161, *p* < .001; Sorensen dissimilarity: Mantel *r* = .262, *p* < .001) (Figure 6). Furthermore, environmental and spatial factors and oribatid mite communities were significantly correlated (Table S5).

The Model 1 VPA (geographic coordinate‐based distances) based on abundance data across nine elevations showed that local, climate, and spatial variables jointly explained 32.1% of oribatid mite community variation (Table 1). Specifically, the pure effects of these three components explained 3.9% (*p* = .001), 1.9% (*p* = .003), and 8.8% (*p* = .001) of community variation, respectively. When presence/absence data were used in VPA, the results followed a similar pattern (Table 1). Consistent with the results of Model 1, Model 2 (elevation‐based distances) based on abundance data, identified pure effects of local (3.9%), climate (1.8%), and elevation (8.8%) components on oribatid mite community variation, jointly explained 32.1% of the total variation (Table 1). The analysis based on presence/absence data showed similar results (Table 1).

**TABLE 1:** Results of variation partitioning analysis for soil oribatid mite communities based on abundance and presence and absence data.

Fractions	Model 1 (geographic coordinate‐based distances)				Model 2 (elevation‐based distances)			
	Abundance		Presence/absence		Abundance		Presence/absence	
	*R* ^2^_adj_	*p*	*R* ^2^_adj_	*p*	*R* ^2^_adj_	*p*	*R* ^2^_adj_	*p*
Local	.173	.001***	.135	.001***	.173	.001***	.135	.001***
Climate	.117	.001***	.115	.001***	.117	.001***	.115	.001***
Spatial	.253	.001***	.180	.001***	.254	.001***	.180	.001***
Local/(Climate + Spatial)	.039	.001***	.025	.003**	.039	.002**	.025	.003**
Climate/(Local + Spatial)	.019	.003**	.040	.001***	.018	.009**	.040	.001***
Spatial/(Local + Climate)	.088	.001***	.064	.001***	.088	.001***	.064	.001***
Local∩Climate∩Spatial	.047	—	.063	—	.047	—	.063	—
Residuals	.679	—	.753	—	.679	—	.753	—

*Note*: The table shows the percentage of variation explained for Model 1 (community versus geographic coordinate‐based distances) and Model 2 (community versus elevation‐based distances). Local/(Climate + Spatial), pure local variation; Climate/(Local + Spatial), pure climate variation; Spatial/(Local + Climate), pure spatial variation obtained from geographic coordinates and elevation data, for Models 1 and 2 respectively; Local∩Climate∩Spatial, shared environmental and spatial component. *R* ^2^ was adjusted for the percentage variation. The complete statistics for the three explanatory factors and each model are provided in Excel spreadsheets. ** represents *p* < .01 and *** represents *p* < .001.

**FIGURE 7:**
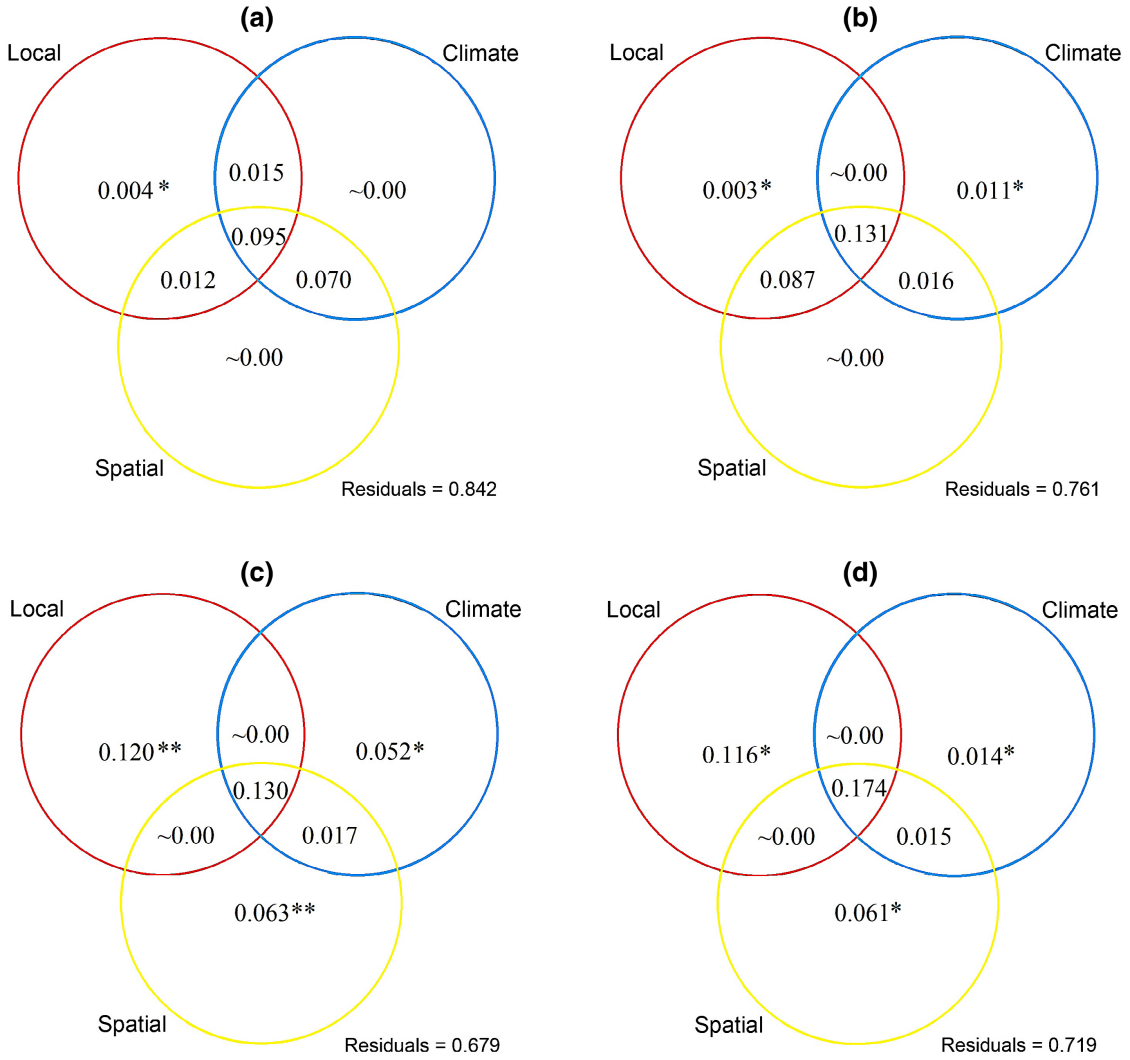
Variation partitioning analysis results showing the percentages of explained variation by local (soil properties), climate (MAT and MAP), and spatial factors (PCNMs) on oribatid mite assembly across four vegetation zones (a–d). (a) mixed coniferous and broad‐leaved forests; (b) mixed coniferous forests; (c) birch forests; and (d) alpine tundra. The complete statistics for the three explanatory factors and each model are provided in Excel spreadsheets. * represents *p* < .05 and ** represents *p* < .01.

The VPA based on abundance data revealed that local, climatic, and spatial factors combined explained 15.8%, 23.9%, 32.1%, and 28.1% (from low to high elevations) of oribatid mite community variation across the four vegetation zones, respectively (Figure 7). In mixed coniferous and broad‐leaved forests, the pure effects of climatic (*F* = 0.681, *p* = .898) and spatial factors (*F* = 0.934, *p* = .614) were small (Figure 7a), while in mixed coniferous forests, the pure effects of climatic factors explained 1.1% of oribatid mite community variation, while the pure effects of spatial factors (*F* = 0.957, *p* = .306) remained small (Figure 7b). In birch forests, the pure effects of climatic, and spatial factors contributed 5.2 and 6.3% to mite community assembly, respectively (Figure 7c). Also, in the alpine tundra, the pure effects of climatic and spatial factors accounted for 1.4% and 6.1% of oribatid mite community compositional variation (Figure 7d). Detailed information on the three explanatory factors and associated models can be found in the accompanying Excel spreadsheets.

## 4 DISCUSSION

### 4.1 Oribatid mite distribution pattern and dominant factors

Others have reported a similar decline in oribatid mite diversity along elevational gradients in various regions, including the eastern slope of the Andes in Mount Kinabalu (Hasegawa et al., 2006), the Reserva Biologica San Francisco Forest (Illig et al., 2010), the Western Lesser Caucasus Mountains (Mumladze et al., 2015), and southern Ecuador (Marian et al., 2018). Soil fauna, due to their strong sensitivity to environmental stressors and their particular susceptibility to perturbations, are often less diverse in high‐altitude communities, compared to their lowland counterparts (Nielsen et al., 2015). While soil oribatid mite community diversity varies across mountain ranges, it typically declines with increasing elevation or follows an unimodal distribution (Bokhorst et al., 2018; Liu, Liu, et al., 2023; Liu, Wu, et al., 2023). Nonetheless, mechanisms underlying these observed patterns differ substantially, possibly due to a synergistic interplay of multiple environmental variables or other unidentified factors associated with oribatid mites (Shen et al., 2020). Two primary categories of scale‐dependent environmental factors could shape soil biodiversity along elevational gradients: (1) regional‐scale factors, such as temperature (Gebert et al., 2020), precipitation (Yin et al., 2017), and evolutionary constraints (Xie, Chen, et al., 2022); and (2) local‐scale abiotic factors, including soil pH (González et al., 2007), soil chemical characteristics (Xie, Sun, et al., 2022), and nutrient availability (Devetter et al., 2017; Illig et al., 2010). Our Changbai Mountain study corroborates these influences while highlighting the essential role of spatial interactions in shaping community structure.

The joint effects of local, climate, and spatial factors consistently provided the best explanation for oribatid mite community compositional variation with elevation and across four vegetation zones on Changbai Mountain. Moreover, correlation analysis showed oribatid mite community dissimilarity increased significantly with geographic, elevation, and environmental distance. The observed distance‐decay relationships further emphasized that both environmental and spatial processes were responsible for oribatid mite community assembly (Hypothesis 1; Gumiere et al., 2016; Guo et al., 2020). This implies that both dispersal constraints and ecological niche processes induced by topographical barriers and pronounced environmental heterogeneity at larger spatial scales jointly shape oribatid mite community structure. A complex interplay of historical, spatial, biotic, and abiotic temporal factors may best explain the absence of consistent biodiversity features along elevational gradients (Tsianou & Kallimanis, 2020; Wang, Hu, et al., 2022; Wang, Zhong, et al., 2022). The balance between deterministic and stochastic processes offers a credible rationale for the genesis and sustenance of spatiotemporal biodiversity patterns, even along elevational gradients (Wang, Hu, et al., 2022; Wang, Zhong, et al., 2022). This appears especially pertinent for ecosystems subjected to severe and fluctuating conditions, where discerning niche and stochastic processes prove challenging (Quenta Herrera et al., 2018).

### 4.2 The contribution of local and climate factors

Soil oribatid mites are highly responsive to soil attributes, as shown by our finding that soil TP and NH_4_^+^‐N were crucial predictors of oribatid mite communities along the elevational gradient (Devetter et al., 2017; Xie, Sun, et al., 2022). Furthermore, the influence of local habitat features remained consistent across nine elevations and four representative vegetation zones on Changbai Mountain. There is a positive feedback relationship between soil TP and NH_4_^+^‐N, and soil biodiversity (Delgado‐Baquerizo et al., 2017; Guo et al., 2020). As elevation increases, a decrease in temperature and primary productivity leads to a loss of soil nutrients, which significantly constrains soil development and affects the upward expansion of oribatid mite communities (Albrecht et al., 2021; Nottingham et al., 2019; Peters et al., 2019; Wang, Hu, et al., 2022; Wang, Zhong, et al., 2022). Moreover, soil characteristics can modulate oribatid mite community elevation response, either independently or synergistically with vegetation, by affecting habitat quality and food availability (Crowther et al., 2019). Although Delgado‐Baquerizo et al. (2019) reported a lack of correlation between temporal variations in aboveground and belowground biodiversity, it remains evident that aboveground resource availability significantly impacts soil biodiversity fluctuations.

Climatic changes along elevational gradients play a crucial role in driving biotic community dynamics and shaping richness and abundance patterns (Montaño‐Centellas et al., 2021; Sundqvist et al., 2013). These findings align with ours, which identified climatic variables (MAT and MAP) as pivotal determinants of oribatid mite community diversity (Mumladze et al., 2015; Xie, Sun, et al., 2022). Across the four vegetation zones on Changbai Mountain, we observed a notable peak in oribatid mite community richness at mid‐elevations, with climatic factors exerting a more pronounced effect at higher elevations (Hypothesis 2). A decreasing oribatid mite richness at higher elevations can be attributed to potent environmental barriers, including deteriorating climatic conditions such as plummeting temperatures and escalating temperature variability, along with diminished habitat complexity, which hinders species survival and colonization (Liu, Liu, et al., 2023; Wang, Hu, et al., 2022; Wang, Zhong, et al., 2022). Conversely, mid‐elevation richness peaks may stem from their species‐rich environments and enhanced productivity (McCain, 2009; Montaño‐Centellas et al., 2021; Quintero & Jetz, 2018). Therefore, the observed decline in oribatid mite diversity primarily results from decreasing temperatures, increasing temperature fluctuations, and reduced habitat complexity and food resources (Montaño‐Centellas et al., 2021).

### 4.3 The spatial filtering contribution

Pure spatial variables accounted for the greatest variation in oribatid mite communities along the elevational gradient. However, the explanatory role of pure spatial variables was low within the same vegetation type at different elevations, indicating that large geographic distances and elevational differences may limit oribatid mite dispersal on a wider scale, with less impact in the same vegetation type at different elevations (Hypothesis 3). Nematode distributions, from global to microscales, are strongly influenced by environmental variables, dispersal mechanisms, and intrinsic population dynamics, including reproduction and competition (Ettema & Wardle, 2002). Dispersal limitation can significantly affect the structure of soil fauna communities, despite their passive dispersal tendencies (Liu et al., 2019). Ptatscheck et al. (2018) documented that vectors such as wind, water, insects, birds, and plants facilitated oribatid mite dispersal over extensive geographical regions. Oribatid mites limited dispersal ability and specific microhabitat requirements intensify interspecific competition for constrained resources at higher elevations (He et al., 2022; Montaño‐Centellas et al., 2020). Besides geographical distance, elevation also significantly influences oribatid mite community variation. Extreme environmental conditions at high altitudes, such as reduced oxygen pressure, low temperatures, and elevated ultraviolet radiation, restrict soil organism distributions (Liu, Liu, et al., 2023; Quenta Herrera et al., 2018). We found a direct elevation impact on oribatid mite communities, with marked reductions in abundance and species richness at higher altitudes.

Environmental and spatial factors contributed to oribatid mite community assembly, with their relative importance fluctuating across vegetation zones. However, a considerable portion of community variation remained unexplained by our variables. This could be attributed to unmeasured variables, species interactions, or sampling method limitations (Gan et al., 2019; Guo et al., 2020; Zinger et al., 2019). Methodological challenges in distinguishing stochastic, from deterministic niche‐based processes highlight the need for more controlled experiments in future studies (Du et al., 2021; Wang, Hu, et al., 2022; Wang, Zhong, et al., 2022). Furthermore, soil fauna dispersal ability is often characterized by a suite of traits, including body size and dispersal mode (Holmstrup et al., 2018). Therefore, in future studies, we will enhance spatial distribution pattern analysis based on functional traits to further understand soil fauna community assembly processes.

## 5 CONCLUSIONS

Soil properties, climate, and spatial filtering significantly impacted oribatid mite communities along an elevational gradient, with their relative importance varying significantly among vegetation zones. The joint effects of local, climate, and spatial variables explained the majority of the observed variance in oribatid mite community composition. As elevation increased, environmental harshness and spatial limitations intensified, while local factor effects on oribatid mite communities remained pronounced. The relative contributions of environmental and spatial filters in shaping oribatid mite communities across the elevation gradient and vegetation types confirmed their explanatory effects on elevational biodiversity trends and community assembly. We provide scientific evidence and empirical support for biodiversity conservation within the Changbai Mountain ecosystem under climate change.

## AUTHOR CONTRIBUTIONS

**Dandan Liu:** Conceptualization (lead); formal analysis (lead); investigation (lead); methodology (lead); supervision (equal); validation (equal); writing – original draft (lead); writing – review and editing (lead). **Haitao Wu:** Conceptualization (equal); funding acquisition (supporting); investigation (supporting); project administration (supporting); resources (supporting); supervision (supporting); visualization (supporting); writing – original draft (equal); writing – review and editing (equal).

## CONFLICT OF INTEREST STATEMENT

The authors declared that they have no conflicts of interest in this work.

## Supporting information


Appendix S1.

Appendix S2.

## Data Availability

The datasets analyzed during the current study are available in the Zenodo repository at https://doi.org/10.5281/zenodo.10429762.
