# Supplementary material for: The joint effects of local, climatic, and spatial variables determine soil oribatid mite community assembly along a temperate forest elevational gradient
Source: Ecol Evol. 2024 Jul 3;14(7):e11590. doi: 10.1002/ece3.11590 (PMC11222168; doi:10.1002/ece3.11590)
Supplement: Supplementary file 1 — Appendix S1. [file ECE3-14-e11590-s002.docx]

Supplementary materials

Title: The joint effects of local, climatic, and spatial variables determine soil oribatid mite community assembly along a temperate forest elevational gradient

Dandan Liu^1^, Haitao Wu^1,^ *

^1^ Key Laboratory of Wetland Ecology and Environment, Northeast Institute of Geography and Agroecology, Chinese Academy of Sciences, 4888 Shengbei Street, Changchun, Jilin 130012, China.

**Correspondence**

Haitao Wu, Key Laboratory of Wetland Ecology and Environment, Northeast Institute of Geography and Agroecology, Chinese Academy of Sciences, 4888 Shengbei Street, Changchun, Jilin 130012, China.

Email: [wuhaitao@iga.ac.cn](mailto:wuhaitao@iga.ac.cn)

Table S1 Summary of the main characteristics of sampling sites.

| Forest type | Longitude | Latitude | Elevation (m) | MAT (℃) | MAP (mm) | Dominant tree species | Soil type |
| --- | --- | --- | --- | --- | --- | --- | --- |
| Mixed coniferous and broad-leaved forests | 128°16′41″ | 42°31′6″ | 600 | 3.15 | 655 | *Pinus koraiensis*; *Quercus mongolica*; *Fraxinus mandshurica*; *Phryma leptostachya; Valeriana amurensis* | Albi-Boric Argosols |
|  | 128°5′46″ | 42°24′8″ | 800 | 2.81 | 688 |  |  |
|  | 128°9′4″ | 42°14′7″ | 1000 | 1.51 | 708 |  |  |
| Mixed coniferous forests | 128°7′42″ | 42°8′27″ | 1200 | 0.55 | 745 | *Picea jezoensis; Abies nephrolepis; Lonicera caerulea*; *Acer ukurunduense; Calamagrostis angustifolia* | Bori-Udic Cambosols |
|  | 128°6′35″ | 42°7′15″ | 1400 | 0.05 | 760 |  | Umbri-Gelic Cambosols |
|  | 128°4′28″ | 42°5′12″ | 1600 | -1.07 | 799 |  |  |
| Birch forests | 128°3′51″ | 42°3′54″ | 1800 | -2.3 | 873 | *Betula ermanii*; *Rhododendron aureum; Vaccinium uliginosum; Lonicera caerulea* | Permi-Gelic Cambosols |
|  | 128°4′0″ | 42°3′26″ | 2000 | -3.07 | 913 |  |  |
| Alpine tundra | 128°3′57″ | 42°2′26″ | 2200 | -4.17 | 955 | *Vaccinium uliginosum*; *Dryas octopetala L.; Rhododendron aureum; Saussurea tomentosa* | Permafrost cold Cambisols |

Table S2 Abundance (individuals per m^2^) of soil oribatid mite across the nine elevations at Changbai Mountains.

| Family | Specie | 600 m | 800 m | 1000 m | 1200 m | 1400 m | 1600 m | 1800 m | 2000 m | 2200 m |
| --- | --- | --- | --- | --- | --- | --- | --- | --- | --- | --- |
| Achipteriidae Thor | *Achipteria coleoptrata* (Linnaeus, 1758) | 5010 | 3892 | 0 | 679 | 0 | 0 | 271 | 0 | 11104 |
| Astegistidae Balogh | *Birsteinius neonominatus* (Subías, 2004) | 1625 | 6369 | 0 | 170 | 0 | 5265 | 542 | 0 | 0 |
|  | *Birsteinius* sp. | 135 | 0 | 0 | 0 | 0 | 2718 | 271 | 2979 | 0 |
|  | *Cultroribula bicultrata* (Berlese, 1905) | 0 | 0 | 0 | 0 | 510 | 679 | 0 | 406 | 0 |
| Brachychthoniidae Thor | *Liochthonius sellnicki* (Thor, 1930) | 0 | 0 | 944 | 4076 | 0 | 679 | 542 | 0 | 5687 |
|  | *Synchthonius elegans* (Forsslund, 1956) | 0 | 118 | 0 | 8832 | 0 | 2208 | 542 | 0 | 0 |
| Carabodidae Koch | *Carabodes labyrinthicus* (Michael, 1879) | 0 | 0 | 0 | 170 | 0 | 0 | 0 | 0 | 0 |
| Cepheusidae Berlese | *Cepheus cepheiformis* (Nicolet, 1855) | 542 | 4246 | 118 | 0 | 0 | 0 | 0 | 135 | 0 |
| Ceratoppiidae Grandjean | *Ceratoppia bipilis* (Hermann, 1804) | 0 | 590 | 590 | 1529 | 849 | 849 | 0 | 1219 | 0 |
|  | *Parapyroppia* sp. | 0 | 0 | 0 | 0 | 0 | 0 | 406 | 0 | 0 |
| Ceratozetidae Jacot | *Ceratozetes gracilis* (Michael, 1884) | 271 | 2831 | 4482 | 679 | 16306 | 2548 | 10427 | 14218 | 5281 |
|  | *Trichoribates berlesei* (Jacot, 1929) | 3521 | 6605 | 4836 | 13928 | 4246 | 3737 | 4604 | 6229 | 2031 |
|  | *Trichoribates* sp. | 406 | 12385 | 12267 | 170 | 13588 | 23609 | 135 | 135 | 1490 |
| Crotoniidae Thorell | *Camisia biurus* (Koch, 1839) | 0 | 0 | 0 | 679 | 0 | 1868 | 0 | 0 | 0 |
|  | *Camisia solhoeyi* Colloff, 1993 | 0 | 590 | 118 | 1019 | 170 | 679 | 5010 | 3927 | 0 |
|  | *Heminothrus longisetosus* (Willmann, 1925) | 0 | 1062 | 236 | 15626 | 340 | 4586 | 135 | 0 | 0 |
|  | *Heminothrus yamasakii* Aoki, 1958 | 9072 | 2595 | 0 | 0 | 0 | 0 | 0 | 0 | 0 |
|  | *Platynothrus peltifer* (Koch, 1839) | 2844 | 826 | 0 | 17665 | 8832 | 56051 | 1760 | 7989 | 0 |
| Damaeidae Berlese | *Dyobelba biclavata* (Wang & Norton, 1993) | 0 | 1887 | 0 | 340 | 0 | 1019 | 1490 | 1625 | 0 |
|  | *Epidamaeus* sp. | 3385 | 3185 | 4718 | 11380 | 2548 | 2038 | 6364 | 1896 | 0 |
|  | *Porobelba spinosa* (Sellnick, 1920) | 1760 | 4246 | 1533 | 12739 | 1189 | 2548 | 1219 | 3656 | 271 |
|  | *Protodamaeus barbatus* (Choi, 1997) | 1083 | 0 | 0 | 0 | 0 | 0 | 0 | 0 | 0 |
|  | *Tokukobelba compta* (Kulczynski, 1902) | 7718 | 5662 | 2949 | 9851 | 2887 | 3907 | 1490 | 542 | 4469 |
| Eniochthoniidae Grandjean | *Eniochthonius minutissimus* (Berlese, 1903) | 1760 | 5308 | 13093 | 24798 | 19363 | 9172 | 135 | 135 | 0 |
| Eremaeidae Oudemans | *Eremaeus borealis* Wen, 1988 | 542 | 354 | 472 | 0 | 0 | 0 | 0 | 0 | 0 |
| Eulohmanniidae Grandjean | *Eulohmannia ribagai* (Berlese, 1910) | 406 | 118 | 2005 | 0 | 170 | 679 | 1083 | 0 | 5010 |
| Euphthiracaridae Jacot | *Acrotritia hauseri* (Mahunka, 1991) | 271 | 4364 | 1533 | 4756 | 3567 | 4756 | 0 | 135 | 0 |
| Galumnidae Jacot | *Acrogalumna* sp. | 2437 | 118 | 0 | 2208 | 170 | 849 | 0 | 0 | 0 |
| Gustaviidae Oudemans | *Gustavia microcephala* (Nicolet, 1855) | 271 | 3657 | 0 | 0 | 0 | 0 | 0 | 0 | 0 |
| Haplozetidae Grandjean | *Incabates* sp. | 0 | 0 | 0 | 340 | 1359 | 0 | 0 | 0 | 0 |
| Hermanniellidae Grandjean | *Hermanniella granulata* (Nicolet, 1855) | 812 | 118 | 0 | 0 | 0 | 510 | 0 | 0 | 0 |
| Humerobatidae Grandjean | *Diapterobates humeralis* (Hermann, 1804) | 1354 | 0 | 0 | 1189 | 1529 | 170 | 406 | 0 | 0 |
|  | *Diapterobates pusillus cuspidatus* Choi, 1986 | 0 | 7313 | 944 | 0 | 1189 | 170 | 0 | 0 | 0 |
| Hypochthoniidae Berlese | *Hypochthonius rufulus* Koch, 1835 | 2167 | 4246 | 1062 | 0 | 0 | 170 | 135 | 6906 | 0 |
| Liebstadiidae Balogh | *Liebstadia similis* (Michael, 1888) | 2302 | 472 | 354 | 510 | 170 | 170 | 542 | 24509 | 271 |
| Malaconothridae Berlese | *Malaconothrus pygmaeus* Aoki, 1969 | 135 | 1769 | 0 | 0 | 0 | 1019 | 1219 | 0 | 0 |
| Nanhermanniidae Sellnick | *Nanhermannia nana* Nicolet, 1855 | 948 | 2831 | 1062 | 1868 | 679 | 1868 | 10291 | 4062 | 0 |
| Nothridae Berlese | *Nothrus anauniensis* Canestrini et Fanzago, 1877 | 1354 | 118 | 236 | 0 | 0 | 170 | 0 | 0 | 0 |
|  | *Nothrus silvestris* Nicolet, 1855 | 0 | 236 | 236 | 1019 | 510 | 0 | 5010 | 0 | 0 |
| Oppiidae Sellnick | *Lauroppia neerlandica* (Oudemans, 1900) | 23020 | 25478 | 49068 | 398471 | 103439 | 63355 | 102234 | 88422 | 10833 |
| Oribatellidae Jacot | *Lepidozetes dashidorzsi* Balogh et Mahunka, 1965 | 0 | 118 | 0 | 0 | 0 | 0 | 0 | 0 | 0 |
|  | *Oribatella linjiangensis* Gao et Wen, 1992 | 0 | 0 | 236 | 0 | 0 | 0 | 0 | 0 | 0 |
| Oribatulidae Thor | *Oribatula tibialis* (Nicolet, 1855) | 135 | 472 | 0 | 0 | 0 | 0 | 0 | 542 | 0 |
|  | *Phauloppia sakamorii* (Aoki, 1970) | 0 | 0 | 0 | 510 | 0 | 0 | 0 | 135 | 0 |
|  | *Zygoribatula exilis* (Nicolet, 1855) | 0 | 0 | 0 | 170 | 0 | 0 | 0 | 0 | 0 |
| Parhypochthoniidae Grandjean | *Parhypochthonius aphidinus* Berlese, 1904 | 3927 | 0 | 2595 | 0 | 0 | 0 | 0 | 0 | 0 |
| Perlohmanniidae Grandjean | *Perlohmannia gigantea* (Aoki, 1960) | 0 | 236 | 0 | 0 | 0 | 0 | 0 | 0 | 0 |
| Phenopelopidae Petrunkevitch | *Eupelops contaminatus* Choi, 1986 | 271 | 1180 | 0 | 170 | 0 | 0 | 0 | 0 | 0 |
| Phthiracaridae Perty | *Austrophthiracarus inusitatus* (Niedbała, 1983) | 8260 | 5190 | 4600 | 679 | 1359 | 1359 | 0 | 3114 | 0 |
| Podopterotegaeidae Piffl | *Podopterotegaeus tectus* Aoki, 1969 | 0 | 0 | 118 | 510 | 0 | 340 | 135 | 0 | 0 |
| Protoribatidae Balogh | *Protoribates capucinus* Berlese, 1908 | 4198 | 9082 | 0 | 0 | 0 | 0 | 0 | 0 | 0 |
|  | *Protoribates lophothrichus* (Berlese, 1904) | 2437 | 2005 | 354 | 0 | 0 | 2718 | 0 | 0 | 0 |
|  | *Protoribates oblongus* (Ewing, 1909) | 135 | 0 | 0 | 0 | 0 | 0 | 0 | 0 | 135 |
| Punctoribatidae Thor | *Punctoribates insignis* Berlese, 1910 | 812 | 0 | 0 | 0 | 0 | 0 | 0 | 0 | 0 |
| Suctobelbidae Jacot | *Allosuctobelba grandis* (Paoli, 1908) | 0 | 118 | 0 | 0 | 0 | 170 | 271 | 0 | 0 |
| Tectocepheidae Grandjean | *Tectocepheus velatus* (Michael, 1880) | 8666 | 24416 | 8257 | 94947 | 18514 | 37877 | 35206 | 49695 | 2302 |
| Tenuialidae Jacot | *Hafenrefferia acuta* Aoki, 1966 | 135 | 472 | 0 | 0 | 0 | 170 | 0 | 0 | 0 |
| Trhypochthoniidae Willmann | *Trhypochthonius tectorum* (Berlese, 1896) | 7177 | 2005 | 1180 | 0 | 340 | 0 | 0 | 0 | 0 |
| Xenillidae Woolley | *Xenillus tegeocranus* (Hermann, 1804) | 0 | 236 | 0 | 0 | 0 | 0 | 0 | 0 | 0 |

Table S3 Spearman's correlations between the abundance of dominant oribatid mite species and environmental variables. Values in bold indicate significant correlations (*P* < 0.05).

|  | TP | MAT | Elevation | NH_4_^+^-N | MAP | pH | TC | TN | NO_3_^-^-N | SM | AP | C/N | DOC |
| --- | --- | --- | --- | --- | --- | --- | --- | --- | --- | --- | --- | --- | --- |
| *Acrotritia hauseri* | 0.026 | **0.338** | **-0.336** | **0.437** | **-0.338** | 0.196 | **0.365** | 0.163 | 0.017 | 0.094 | **0.414** | 0.135 | **0.554** |
| *Achipteria coleoptrata* | 0.165 | 0.082 | -0.067 | -0.103 | -0.082 | **0.272** | -0.212 | -0.060 | **-0.278** | -0.116 | 0.029 | **-0.364** | -0.136 |
| *Heminothrus longisetosus* | -0.160 | 0.123 | -0.108 | 0.172 | -0.123 | -0.007 | 0.093 | -0.054 | 0.129 | -0.069 | **0.375** | 0.162 | 0.201 |
| *Nanhermannia nana* | **0.301** | -0.011 | -0.011 | 0.109 | 0.011 | -0.135 | 0.175 | **0.284** | **0.383** | 0.229 | -0.012 | -0.166 | 0.034 |
| *Austrophthiracarus inusitatus* | **0.374** | **0.521** | **-0.523** | **0.391** | **-0.521** | **0.463** | 0.126 | **0.267** | -0.224 | **0.343** | -0.095 | **-0.233** | 0.020 |
| *Porobelba spinosa* | -0.041 | 0.164 | -0.149 | 0.118 | -0.164 | 0.065 | 0.110 | -0.007 | 0.052 | -0.123 | 0.112 | 0.125 | 0.148 |
| *Liebstadia similis* | 0.040 | -0.133 | 0.134 | **-0.261** | 0.133 | -0.064 | -0.087 | -0.092 | 0.205 | 0.085 | **-0.393** | 0.053 | -0.208 |
| *Epidamaeus* sp. | -0.036 | **0.374** | **-0.371** | 0.111 | **-0.374** | 0.037 | 0.056 | -0.081 | 0.074 | -0.018 | -0.126 | 0.165 | 0.075 |
| *Tokukobelba compta* | 0.067 | **0.324** | **-0.321** | 0.149 | **-0.324** | **0.261** | -0.046 | -0.027 | -0.175 | -0.060 | 0.168 | -0.181 | -0.021 |
| *Trichoribates berlesei* | -0.072 | 0.020 | -0.012 | 0.097 | -0.020 | -0.067 | 0.073 | 0.003 | 0.113 | -0.080 | 0.088 | 0.098 | 0.121 |
| *Ceratozetes gracilis* | 0.021 | **-0.373** | **0.381** | -0.045 | **0.373** | **-0.286** | 0.063 | 0.064 | 0.131 | -0.049 | -0.052 | 0.020 | -0.001 |
| *Trichoribates* sp. | 0.077 | 0.169 | -0.161 | **0.478** | -0.169 | **0.267** | **0.396** | 0.212 | 0.068 | 0.220 | **0.566** | **0.250** | **0.440** |
| *Eniochthonius minutissimus* | -0.074 | **0.436** | **-0.433** | **0.517** | **-0.436** | 0.232 | **0.321** | 0.109 | -0.106 | 0.137 | **0.316** | 0.176 | **0.385** |
| *Platynothrus peltifer* | -0.050 | -0.061 | 0.062 | 0.010 | 0.061 | -0.030 | 0.020 | -0.138 | 0.169 | -0.224 | 0.132 | 0.168 | 0.097 |
| *Tectocepheus velatus* | -0.104 | -0.094 | 0.091 | -0.012 | 0.094 | -0.202 | 0.101 | -0.035 | **0.366** | -0.107 | -0.007 | 0.121 | 0.097 |
| *Lauroppia neerlandica* | **-0.238** | -0.068 | 0.088 | -0.095 | 0.068 | **-0.325** | 0.136 | -0.047 | 0.168 | -0.072 | -0.044 | **0.272** | -0.050 |

Table S4 Spearman's correlations between the diversity of oribatid mite species and environmental variables. Values in bold indicate significant correlations (*P* < 0.05).

|  | TP | MAT | Elevation | NH_4_^+^-N | MAP | pH | TC | TN | NO_3_^-^-N | SM | AP | C/N | DOC |
| --- | --- | --- | --- | --- | --- | --- | --- | --- | --- | --- | --- | --- | --- |
| Abundance | **0.135** | **0.231** | **0.269** | **0.143** | **0.217** | **0.094** | **0.106** | 0.063 | **0.132** | **0.122** | 0.045 | **0.126** | **0.153** |
| Richness | **0.132** | **0.358** | **0.372** | **0.332** | **0.338** | **0.163** | **0.160** | **0.184** | 0.064 | 0.035 | 0.012 | 0.001 | **0.199** |
| Shannon | **0.319** | **0.139** | **0.143** | **0.217** | **0.085** | **0.235** | **0.158** | **0.279** | 0.022 | **0.118** | 0.025 | 0.074 | **0.166** |

Table S5 Mantel and partial Mantel tests for the correlation between oribatid mite community dissimilarity and environmental and spatial factors using Pearson’s coefficient.

| Effects of | Controlling for | Bray-Curtis | | Sorensen | |
| --- | --- | --- | --- | --- | --- |
|  |  | *r* | *P* | *r* | *P* |
| Environmental |  | 0.161 | **0.001** | 0.262 | **<0.001** |
| Spatial (geographic distances) |  | 0.203 | **<0.001** | 0.356 | **<0.001** |
| Spatial (elevation distances) |  | 0.203 | **<0.001** | 0.357 | **<0.001** |
| Environmental | Spatial (geographic distances) | 0.002 | 0.457 | -0.030 | 0.685 |
| Environmental | Spatial (elevation distances) | 0.002 | 0.462 | -0.032 | 0.708 |
| Spatial (geographic distances) | Environmental | 0.126 | **0.004** | 0.252 | **0.001** |
| Spatial (elevation distances) | Environmental | 0.126 | **0.008** | 0.254 | **0.001** |


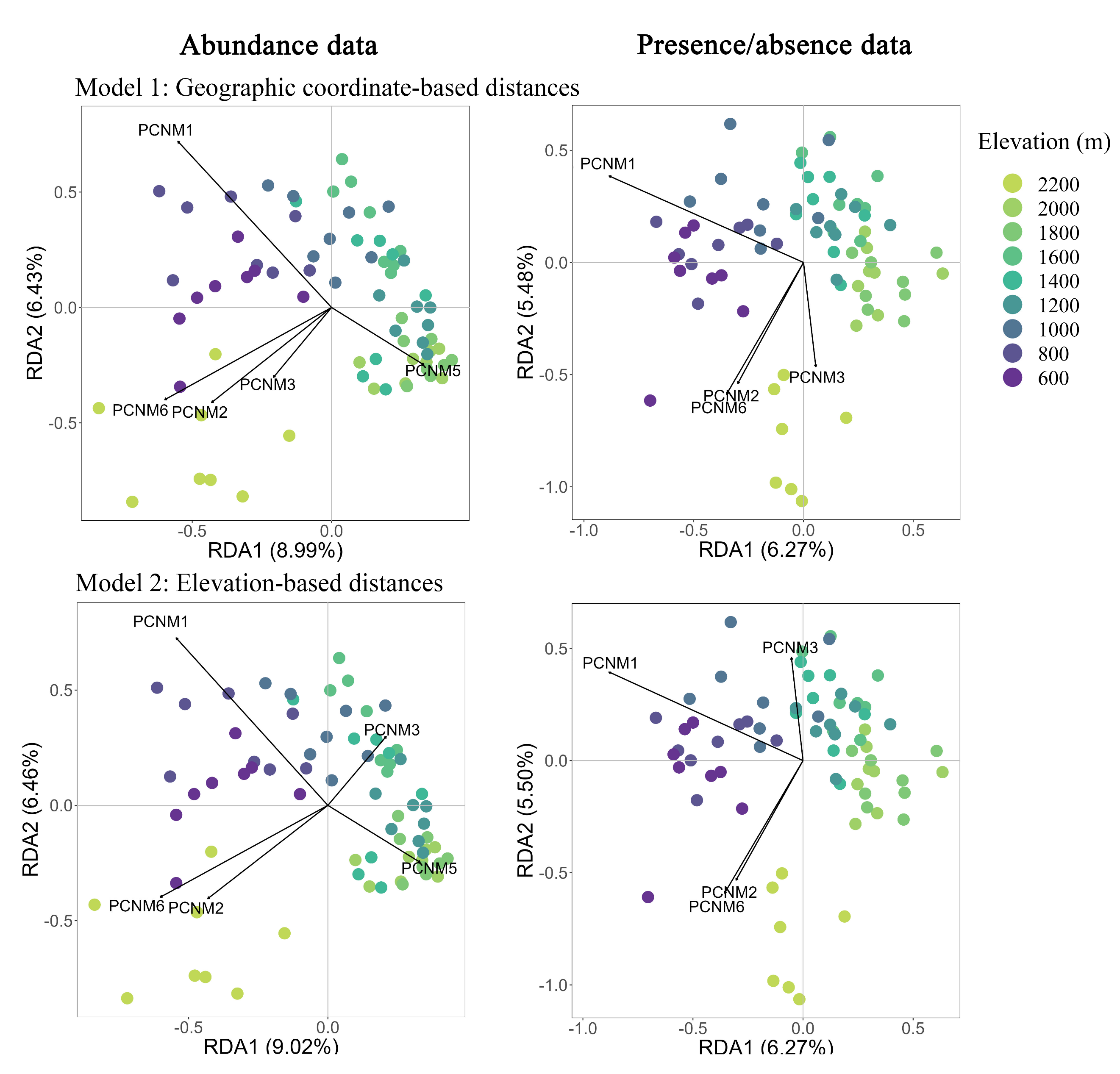


Figure S1 RDA ordinations showing the soil oribatid mite community composition based on abundance data or presence/absence data, in relation to significant (*P* < 0.05) spatial variables for Model 1 (geographic coordinate-based distances) and Model 2 (elevation-based distances).


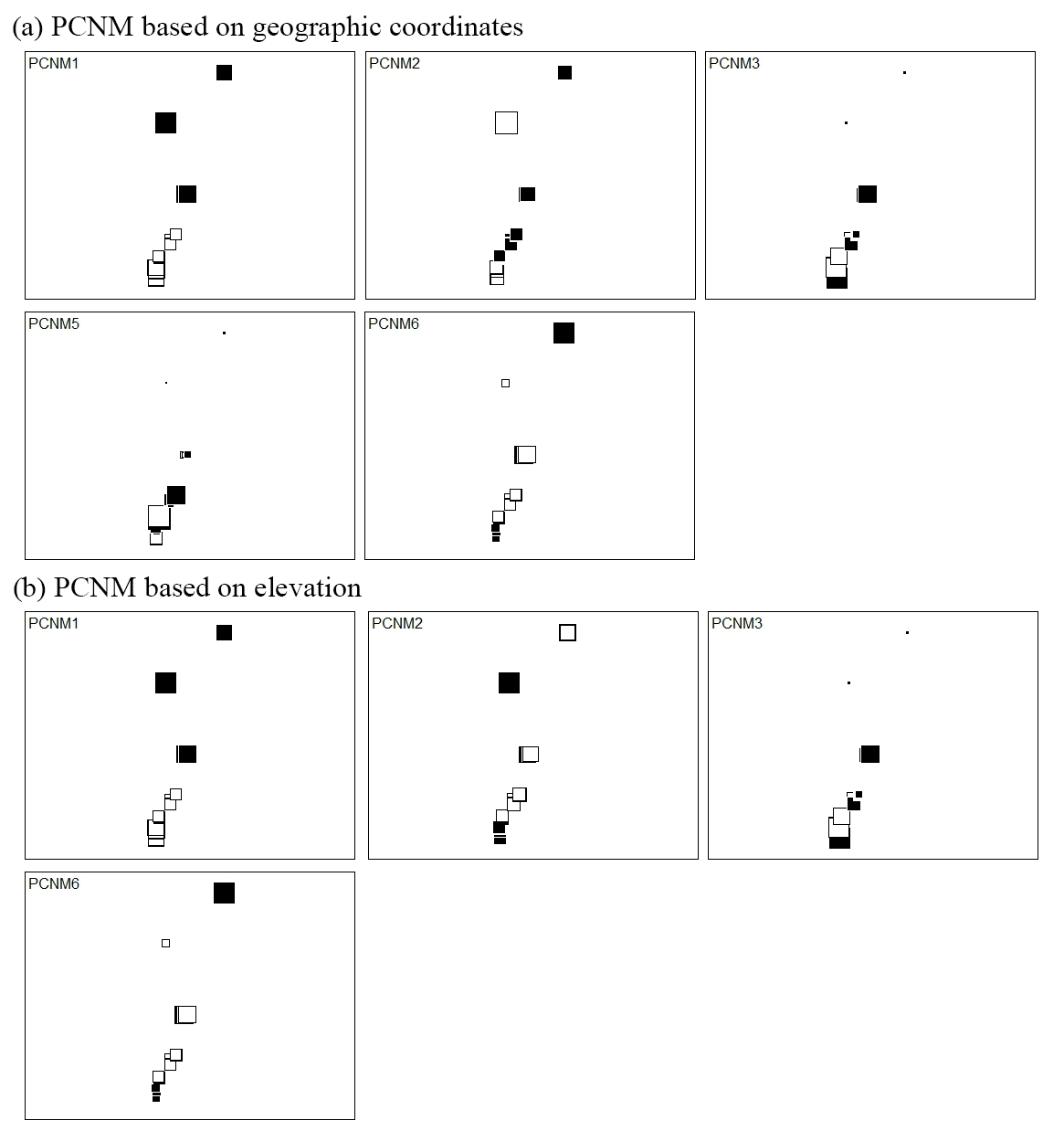


Figure S2 Construction of PCNM map based on the geographical coordinates and elevation of each sampling site.


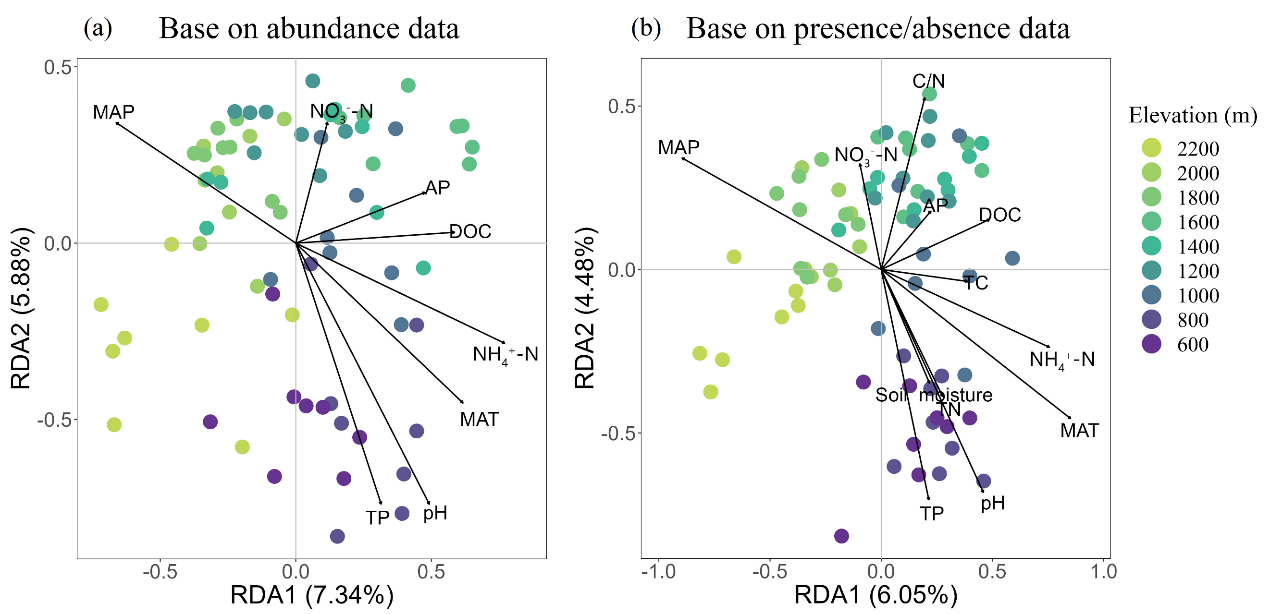


Figure S3 RDA ordinations showing the soil oribatid mite community composition based on (a) abundance data and (b) presence/absence data in relation to significant (*P* < 0.05) environmental variables.
